# The Nature of Anti-Asian American Xenophobia during the Coronavirus Pandemic: A Preliminary Exploration into Envy as a Key Motivator of Hate

**DOI:** 10.3390/bs11110158

**Published:** 2021-11-19

**Authors:** Daisuke Akiba, Ana Sofia Velazquez Lopez, Mari Hirano

**Affiliations:** 1 Queens College & The Graduate Center, The City University of New York, Queens, NY 11367, USA; 2 The Warren Alpert Medical School, Brown University, Providence, RI 02903, USA; anasofia_velazquez@brown.edu; 3 Office of Labor Health and Welfare, The City of Sapporo, Sapporo 060-8611, Hokkaido, Japan; mari-hirano@hokkaido.med.or.jp

**Keywords:** AAPI, Asian Americans, Asiaphobia, Coronavirus, COVID-19, envy, experiment, hate, racism, xenophobia

## Abstract

**Background**. The current Coronavirus pandemic has been linked to a dramatic increase in anti-Asian American and Pacific Islander (AAPI) hate incidents in the United States. At the time of writing, there does not appear to be any published empirical research examining the mechanisms underlying Asiaphobia during the current pandemic. Based on the stereotype content model, we investigated the idea that ambivalent attitudes toward AAPIs, marked primarily with envy, may be contributing to anti-AAPI xenophobia. **Methods**. Study 1 (N = 140) explored, through a survey, the link between envious stereotypes toward AAPIs and Asiaphobia. Study 2 (N = 167), utilizing autobiographical recall tasks, experimentally induced the affect of envy in order to establish causality between feelings of envy toward AAPIs and Asiaphobia. **Results**. In Study 1, envious stereotypes toward AAPIs were found to be predictive of Asiaphobia and, in Study 2, the inducement of envy led to heightened levels of Asiaphobia. **Conclusions**. The current research provides support for the proposition that, consistent with the stereotype content model, stereotypes and attitudes toward AAPIs marked with ambivalent and envious views, consisting of a mix of perceived competence and lack of “human warmth,” may be fueling Asiaphobia. Implications for potential applications and future research are discussed.

## 1. Introduction

Since the onset of the current Coronavirus pandemic in 2020, thousands of hate incidents against Asian Americans and Pacific Islanders (AAPIs) have been reported, ranging from physical assaults and racist rants to the refusal of services [1,2,3]. Although the American news media have not consistently reported these anti-AAPI hate incidents and, instead, at times treated them as isolated incidents, reports of such episodes have continued to intensify during the second year of the global pandemic [4,5]. In addition to blatant anti-AAPI hate incidents, AAPIs have been grappling with subtler forms of xenophobia which may lead to chronic stress that could potentially compromise their overall health, such as being routinely exposed to anti-AAPI sentiments online, seeing attributions widely made to Asia and its people vis-à-vis the pandemic in the media, or witnessing individuals avoid AAPIs, ostensibly fearing infection [2,6]. It is therefore not surprising that some have characterized the climate of anti-AAPI xenophobia—or *Asiaphobia*—during the Coronavirus pandemic as a pandemic in its own right [6] (In this manuscript, the term, “Asiaphobia,” is used to refer to anti-AAPI xenophobia and related concepts, such as anti-AAPI stereotyping, prejudice, and discrimination).

Why investigate Asiaphobia in the context of the Coronavirus pandemic? From a public health standpoint, the possibility of Asiaphobia adversely affecting the mental health and psychosocial wellbeing of AAPIs has been proposed by a number of scholars since the onset of the Coronavirus pandemic in early 2020 [2,7,8]. In particular, the critical importance of addressing Asiaphobia has been empirically highlighted by Cheah and her colleagues [6], who have released the results of a survey that they conducted as part of their larger research concerning adults of Chinese origin (N = 543) and their children aged between 10 and 18 years (N = 230) residing in the U.S. For both age groups, in-person or online experiences of Coronavirus-related Asiaphobia, perceived Sinophobia in healthcare, and perceived Sinophobia in the media were found to be linked to: (a) compromised overall psychological health; (b) symptoms of anxiety; and (c) depressive symptoms. Needless to say, these consequences may not merely be outcomes and, instead, they may further compromise the health and quality of life among the affected population. Furthermore, it is curious that even vicarious experiences of Asiaphobia (e.g., witnessing other AAPI individuals being victimized), in person or virtually, were also predictive of symptoms of both anxiety and depression. It is therefore reasonable to conclude that Asiaphobia may take a toll on the psychological health of AAPI individuals upon actual victimization or, to a lesser degree, vicarious victimization.

With the adults and children surveyed by Cheah and her team reporting compromised psychological health, ostensibly in direct response to actual and perceived Sinophobia and Asiaphobia [6], scholars and practitioners need to unite to tackle this crisis by developing measures to alleviate the health impact of the Asiaphobia. In order to develop such measures, we must first better understand Asiaphobia’s underlying motives and mechanisms. Here, it may be important to note that, because the distinctions among the Chinese Communist Party, China, the Chinese, and the rest of the Asian nations and peoples often remain largely fuzzy in the perceptions of mainstream U.S. culture, all AAPI individuals are frequently placed into one large category that is erroneously perceived to be homogeneous [8,9]. As such, xenophobia that stems from negative stereotypes and attitudes toward a specific Asian nation or group, such as the Chinese Communist Party, China, or the Chinese, is likely to have a cascading effect, impacting anybody that appears to be AAPI, regardless of national heritage or citizenship status, in the current pandemic [7].

### 1.1. Asiaphobia and the Sociopolitical Circumstances

At the time of writing, empirical investigations probing the etiology and nature of Asiaphobia as they relate to the Coronavirus pandemic seem to be very much in their infancy. Although the mechanisms underlying Asiaphobia in the current pandemic may thus remain largely unknown from an empirical standpoint for now, researchers assert that Coronavirus’s presumed link to Asia and AAPI people—regardless of whether it is warranted or not—has fueled Asiaphobia at the societal level in the United States [2,3,6].

First, many Americans appear to attribute the roots of the current pandemic to the actions of the Chinese government and Chinese people in one way or another [8]. The outbreak of Coronavirus was first reported globally in early 2020, stemming from Wuhan, China, before spreading quickly to other continents, and this appears to have led to the widespread conviction that China is the virus’s presumed place of origin [10]. Moreover, the emergence of the virus has often been attributed in the United States, more specifically, to factors ranging from the substandard hygiene practices in China [11], the unusual dietary habits in China, such as the consumption of wild bats [12], to a suspected conspiracy involving the Chinese Communist Party and its subsidiaries, such as Wuhan’s virological laboratory [13]. Even though there has been no compelling evidence to substantiate any of these claims definitively, each of these suspicions—prevalent in varying degrees at the societal level in the United States—may contribute to a climate where negative beliefs and attitudes toward China and its people are fostered, laying the foundations upon which Asiaphobia may transpire.

Secondly, there has been a prominent political undercurrent in the United States, often spearheaded by former President Donald Trump and members of his administration, reinforcing these beliefs and attitudes regarding China being the “instigator” of the current pandemic. Since Mr. Trump gave a speech in March 2020, during which he referred to Coronavirus as the “Chinese virus,” he, his staff, and his supporters have routinely called the virus “China virus,” “Wuhan virus,” “Asian virus,” and “kung flu” [14], seemingly without differentiating a foreign region and its people from residents of the United States with cultural roots in that region, unlike what other political leaders have done in the past (see [15]). This mindset has been reinforced by an internal memorandum issued by the National Republican Senatorial Committee, in which Republican candidates running for office nationwide were instructed to: (a) blame China and its officials for causing and spreading the virus; and (b) draw the public’s attention away from Mr. Trump when discussing the pandemic by relentlessly attacking China [16]. Although current President Joe Biden signed a hate crimes bill to combat anti-AAPI violence in May 2021 [17], the accusatory political climate against China and Chinese people prevalent in the United States during the Trump era seems to have set the foundation upon which Asiaphobia may be propelled.

Recently, there have been what appear to be cyclical resurgences of infections, often attributed to the reopening of societies across the globe and the transmission of highly infectious variants of the original virus [18,19]. Given this larger backdrop, some suggest that restrictions designed to curtail infections, such as partial or total shelter-in-place orders, curfews, social distancing, the temporary closures of businesses, mask mandates, and off-campus learning, may very well be reinstituted, perhaps intermittently, in the future [20,21,22]. In the U.S., waves of economic hardship have impacted millions, presumably as a result of these measures [23], and millions of Americans are believed to have been facing housing and food instability [24]. Thus, it is reasonable to assert that health-related threats directly linked to Coronavirus, combined with these potentially recurring restrictions and hardships, have been creating stressful and frustrating situations for people from a diverse range of backgrounds [3].

With the mounting frustration and challenges that many Americans have presumably been facing, an increase in xenophobia and hate incidents, perhaps, might not necessarily be entirely unexpected. Historically, situations involving health threats and resulting socioeconomic hardships have led to the scapegoating of the ethnic minority groups who were perceived as responsible for the outbreaks [25]. This is certainly consistent with the heightened levels of Asiaphobia being witnessed in the U.S. during the current pandemic. Little is known, however, about the factors that contribute to individual variability in Asiaphobia, making it unclear why some individuals seem particularly Asiaphobic compared to others. This study aims to demystify the underlying mechanisms of Asiaphobia so as to better understand how the present-day anti-AAPI climate that has accompanied the current pandemic could be alleviated in order to restore safe and peaceful environments for AAPIs.

### 1.2. Envious Stereotypes as a Predictor of Asiaphobia

The works by Susan Fiske, Peter Glick, and their associates on the stereotype content model [26,27,28,29,30] may provide some clues as to why some individuals may display more heightened tendencies of Asiaphobia than others. The model has shown that AAPIs, in the United States, are subject to ethnic stereotypes and prejudice different in nature from those typically faced by other groups of color, such as African Americans and Latinx. AAPIs are often perceived as being more competent and socioeconomically successful than their White counterparts—not to mention other non-White groups—by exceeding all ethnic groups in domains such as education and income [2,29]. With many Americans facing pandemic-related challenges as discussed above, it would be reasonable to theorize that the widely held presupposition of AAPIs being “socioeconomically better off” than other groups can foster a sense of resentment toward AAPIs. At the same time, according to the stereotype content model, AAPIs are frequently stereotyped as cold, unfriendly, and “not at all nice” [30] (p. 880).

This ambivalent combination of perceived competence/socioeconomic success and perceived coldness/unfriendliness has been theorized to result in envy-based anti-AAPI prejudice among non-AAPI Americans, as measured by the Scale of Anti-Asian American Stereotypes (SAAAS) [27]. High scores on this scale signal envious anti-AAPI stereotyping, which, in turn, has been linked to the “othering” of AAPIs, marked with dehumanized, unsympathetic, and discomfort-ridden attitudes—which makes AAPIs easy targets for social exclusion, rejection, and scapegoating [28,30] (It should be noted here that, although the title of the scale emphasizes “stereotypes,” Lin and her colleagues [27], as well as Fiske [26] and Glick [28], extensively refer to the attitudinal dimensions, such as affective components pertaining to envy and ambivalent attitudes toward AAPIs, throughout their manuscripts when discussing what the SAAAS entails). Fiske [26] expands on this notion, based on the stereotype content model, and posits that non-AAPIs who hold envious prejudice against AAPIs may not just be indifferent, but may also experience schadenfreude, whereby they “can’t help smiling a little” when seeing the envied outgroup encounter difficulties (p. 703). Taken together, according to this line of research, “envious Asiaphobia” entails two key consequences. First, it has been linked to the pronounced othering of AAPIs as members of a dehumanized, rejected, and scapegoated outgroup. Second, envious Asiaphobia is marked with indifference and apathy—or even schadenfreude, as discussed above—regarding any hardships AAPIs may face (e.g., AAPIs becoming victims of xenophobic attacks). With the social integration of AAPIs potentially being challenged in such profound ways, it would be reasonable to assert that the current climate of Asiaphobia will entail psychological and physical health consequences [2,6,31].

Hypothetically, had AAPIs been associated with a different pattern of stereotypes (e.g., personable and friendly, but not as competent) in the current pandemic, according to the stereotype content model, perhaps non-AAPI members of the society would have, for example, pitied and sympathized with AAPIs more and/or felt the urge to protect and support them [26] (p. 699). If present, the affective backdrop, consisting of a combination of sympathy and support (albeit with paternalistic overtones), would have perhaps prompted non-AAPI individuals to take action against Asiaphobia more enthusiastically. In reality, however, such movements have only emerged sporadically, typically led by members of the AAPI community [32,33].

As discussed earlier, the United States has recently experienced a climate in which citizens may: (a) continue to face the threat of Coronavirus infection, although it may have been alleviated as more individuals get vaccinated; (b) experience stress and frustrations associated with personal, social, and economic consequences—actual or anticipated—of infection-control measures; (c) be exposed to a variety of information, much of it false, ascribing the origin of the virus to China and its people; and (d) witness government officials and others relentlessly blaming China and Chinese people for the pandemic. Given these considerations, it is reasonable to suggest that there are situational factors that may induce anti-AAPI sentiments in some individuals in the U.S. On the individual level, based on research by Fiske, Glick, and their colleagues discussed above [26,28], it would be reasonable to hypothesize that envious stereotypes concerning AAPIs will be predictive of Asiaphobia during the current pandemic.

Two studies were conducted to establish a relationship between envious anti-AAPI prejudice and Asiaphobia. Study 1, through an exploratory correlational inquiry, sought to examine the degree to which envious prejudice against AAPIs may predict Asiaphobia. Study 2 induced envy toward AAPIs experimentally before measuring Asiaphobia, thereby attempting to establish causality between envy toward AAPIs and the pandemic-related Asiaphobia. The following research questions were addressed through the two studies: (1) To what degree, if at all, does envious stereotyping of AAPIs predict Asiaphobia as it pertains to the current Coronavirus pandemic (Study 1)? and (2) How would the experimentally induced effect of envy be related to Asiaphobia (Study 2)?

## 2. Study 1: Materials and Methods

### 2.1. Overview

Data were collected through online surveys to examine the relationship between: (a) envious prejudice toward AAPIs; and (b) Asiaphobia. An online survey company, Pollfish, was retained to recruit and deliver the survey, primarily because institutionally based participant recruitment during the pandemic proved to be challenging. Of a number of service providers, Pollfish was selected here for two major reasons. First, it gained much publicity after it was identified as the only survey firm to have enabled researchers to accurately predict Brexit [34]. Second, researchers at major research institutions have shown Pollfish’s participants to be comparable to participants in studies by the Pew Research Center and the General Social Survey, and the results obtained through Pollfish to be largely accurate [35]. With 10 million active users, Pollfish has successfully been utilized in social sciences research involving racial stereotypes and ethnic relations [36].

### 2.2. Participants

Adults living in the United States between the ages of 18 and 64 were recruited to participate in this study. Pollfish distributed survey invitations through its third-party digital application partners, who provided nominal incentives to survey completers. It does not, however, release information on specific compensation schedules to researchers and, thus, it is unclear as to how each participant was incentivized. Pollfish executed quality checks on the data obtained and excluded respondents whose responses were assessed to lack integrity, albeit without disclosing the number of excluded cases (Pollfish includes quality check questions that are presumably impossible to miss if participants are paying sufficient attention [e.g., trap and red herring questions], in order to ensure proper participant engagement. Participants are excluded casewise for not answering these questions correctly). Data collection was conducted until the number of respondents whose responses met Pollfish’s quality standards reached 160.

Of these 160 participants, 17 were classified to be AAPI and three were identified as either “mixed” or “other,” signaling that they could potentially be part AAPI. Given the nature of this study, which involves attitudes and beliefs regarding AAPIs as outgroups, these participants were excluded from the analysis. In the end, 140 participants were included in the analysis. Detailed demographic characteristics of the participants are presented in Table 1.

### 2.3. Measures

The survey included two scales: (1) envious stereotypes toward AAPIs; and (2) Coronavirus-related Asiaphobia. The former was a 15-item scale that was developed based on Lin and her associates’ 25-item *Scale of Anti-Asian American Stereotypes* (SAAAS), which measures envious stereotypes toward AAPIs [27]. The original scale included 12 questions regarding AAPIs’ competence and 13 questions on their unsociability, all on a six-point Likert-type scale. Of those, eight *competence* questions with factor loadings above 0.60, as reported by Lin et al. [27], were selected, along with seven *unsociability* questions with the same factor loading cut off, for a total of 15 questions to be included in the current study. The short, 15-item version that we developed here proved to be highly reliable with our current sample (ω = 0.86), and the items are listed in Table 2.

Concurrently, an eight-item Coronavirus Asiaphobia scale was developed to assess the degree to which individuals express and condone Asiaphobia vis-à-vis the current pandemic, and the questions are listed in Table 3.

A two-pronged approach was taken in developing this scale, which consists of eight questions. First, two separate focus group sessions with a total of six AAPI, White, and Latinx professional males and females, mostly attorneys and university faculty in the social sciences between the estimated ages of 30 and 50, were held online, during which they were asked to engage in a thought exercise to identify the attitudes, beliefs, and other “thought characteristics,” in their view, commonly held by individuals that engage in anti-AAPI hate incidents during the current pandemic. The following underlying thematic dimensions were identified during this conversation, specifically targeting AAPIs: (a) AAPIs posing potential risks for infection; (b) AAPIs being responsible for the pandemic; (c) rationalizing Asiaphobia and believing that AAPIs are deserving of it; and (d) apathy, including the failure to recognize Asiaphobia as a problem. Second, based on research on envy as it relates to the stereotype content model [26], it was ensured that the cornerstones of envious ambivalence toward the outgroup—exclusion, rejection, dehumanization, and victim-blaming—were all thematically present within the scale. All eight questions followed the same six-point Likert-type scale as the SAAAS above. This Coronavirus Asiaphobia scale was found to be reasonably reliable (ω=0.76).

### 2.4. Procedure

Participants recruited through Pollfish completed an online survey through Pollfish’s platforms. It should be noted that, in addition to the IRB clearance obtained through the first author’s institution, the survey was subject to Pollfish’s own terms and conditions, which include ethical considerations. The informed consent process was completed through Pollfish’s protocol, with the primary author’s institution-mandated IRB statement presented to participants prior to commencing the survey. This statement contained the author’s contact information, and participants were instructed to contact him with any questions or concerns about the survey. Pollfish provided the basic demographic information on the participants (e.g., race/ethnicity, sex, age bracket, etc.) with the data collected; as such, demographic information was not collected during the survey.

Using Pollfish’s “grouping and shuffling” feature, the sequence of the short version of the SAAAS (SAAAS Short) and the Coronavirus Asiaphobia scale was counterbalanced, with participants randomly receiving either SAAAS Short or the Asiaphobia scale first. In addition, within each scale, the order of presentation of the questions was randomized. Debriefing was not required by the IRB, as the study did not involve any deception or otherwise sensitive procedures.

## 3. Study 1: Results and Discussion

The short version of the measure of envious anti-AAPI stereotypes by Lin and her colleagues [27] contained 15 items, each asked on a six-point Likert-type scale from 0 to 5. It thus had a lowest possible score of 0 (not at all enviously prejudiced) and the highest possible score of 75 (extremely enviously prejudiced). The mean score was relatively modest (M = 36.77; SD = 12.18). Scores on the eight-item Asiaphobia scale (0 = no Asiaphobia; 40 = high Asiaphobia) also yielded modest levels of Asiaphobia (M = 13.46; SD = 7.81). A linear regression analysis revealed that envious AAPI stereotyping was predictive of Asiaphobia (R^2^ = 0.311, F(1, 138) = 62.15, *p* < 0.0001). Analyses involving participants’ demographic backgrounds were not performed, as: (a) the distribution of demographic backgrounds among the participants was highly skewed, although it seems reasonably representative of the population being surveyed; and (b) there are no theoretical reasons to suspect differences among them, according to the stereotype content model.

While Study 1 has successfully demonstrated that envious stereotyping against AAPIs seems to predict higher levels of Asiaphobia in the current pandemic, due to the correlational nature of Study 1, the causal relationship remains unclear. Though interesting and informative, the statistically significant relationship found in this study is not sufficient for drawing causal inferences.

In order to establish the causal relationship between envy toward AAPIs and Coronavirus Asiaphobia, as proposed based on the stereotype content model [30], further investigation is necessary. To this end, Study 2 employed an experimental method to induce envy toward AAPIs through autobiographical recall to explore the causal relationship. It was hypothesized in Study 2 that, based on the stereotype content model [26,30], inducement of envy toward AAPIs will lead to higher scores on the Coronavirus Asiaphobia scale [27].

## 4. Study 2: Materials and Methods

### 4.1. Participants

As was the case in Study 1, adults between the ages of 18 and 64 were recruited, and they completed this experiment online. Participants were randomly assigned to one of the two experimental conditions, and the data collection continued until the number of respondents whose responses met Pollfish’s data quality standards reached 100 in each condition—for a total of 200 participants. Participants received nominal incentives through Pollfish’s partners; however, since Pollfish does not disclose the compensation schedule to researchers, it is unclear as to how each participant was compensated. The participants’ demographic characteristics are described in detail in Table 4.

Of the 200 participants, 16 identified themselves as AAPI. In addition, 17 were identified as either “mixed,” “other,” or “do not wish to answer,” suggesting that these 33 individuals could potentially have AAPI heritage in varying degrees. Since the current study involved attitudes toward AAPIs from non-AAPI perspectives, these 33 participants were excluded from the analysis, resulting in the inclusion of 167 participants in the analysis (80 in Condition 1 and 87 in Condition 2).

### 4.2. Materials and Procedure

Informed consent was obtained through Pollfish’s protocol, and an IRB statement as required by the primary author’s institution was presented to participants as part of the consent process. In this statement, contact information for the first author was presented, so participants could contact him with any questions or concerns. In Study 2, all participants were told that they will be answering an eclectic selection of survey questions on attitudes and beliefs, and each participant was randomly assigned to one of the following two experimental conditions: (1) the inducement of envy toward AAPIs condition; and (2) the inducement of general envy condition. They were subject to the assigned experimental manipulation, whereby envy toward AAPIs was induced in Condition 1, while envy in general, without specifically targeting a racial, ethnic, or any other cultural group, was induced in Condition 2. Specifically, participants in Condition 1 were presented with the following open-ended question first, and they were not permitted to proceed unless they responded open-endedly to this question:

*In this question, we are interested in learning about how the emotion of “envy” may play a role in social relations across cultural groups. Please take a minute or so to think about an occasion when you felt envious of Asian-American people, and briefly describe the situation in the space provided.*

This task was designed to induce envy toward AAPIs through autobiographical recall, applying a method previously validated to be effective in inducing a range of affects [37]. Participants in Condition 2, by contrast, were asked to complete a slightly different task, as follows:

*In this question, we are interested in learning about how the emotion of “envy” may play a role in social relations. Please take a minute or so to think about an occasion when you felt envious of a friend or colleague of yours, and briefly describe the situation in the space provided.*

In this condition, the same affect—envy—was induced, but it was done in reference to the participants’ friends and colleagues without mentioning any specific groups; thus, any emotion of envy that might result from this experimental treatment should not be explicitly linked to particular social groups across participants in this condition. In both, a reminder was included that: (a) their responses will remain confidential; and (b) the anecdote being described could be either the participants’ own first-hand experience or based on something they saw or heard.

While envy was induced in both experimental conditions, participants in Condition 1 were specifically asked to think of AAPIs as the target of their envy while those in Condition 2 were not. While Condition 2 did not preclude the possibility of the participants thinking of their AAPI friends or colleagues during the biographical recall task, we determined that this did not compromise the quality of our data since: (a) it was deemed highly unlikely that the results would be systematically skewed to be reflective of envy toward AAPIs; and (b) none of the participants in Condition 2 mentioned AAPIs or any descriptions explicitly pertaining to them (e.g., an anecdote about a neighbor who is described as AAPI or AAPI-related labels).

Subsequent to the experimental treatment above, the same eight-item Coronavirus Asiaphobia scale used in Study 1 (see Table 3) was administered. As was the case in Study 1, the lowest possible score on this scale was 0 (not at all Asiaphobic) and the highest possible score was 40 (highly Asiaphobic), and the questions were presented in a random order using Pollfish’s “grouping and shuffling” feature. Any differences found in the dependent variable, Asiaphobia, could thus reasonably be attributed to the target specificity of the envy being induced. In addition, at the end of the survey, participants were asked to answer questions regarding the degree to which: (a) the health of the participants or their loved ones has been impacted by Coronavirus; (b) the financial well-being of the participants or their loved ones has been adversely affected by the pandemic; and (c) the participants regularly interact with their AAPI friends, neighbors, or colleagues—all on the same Likert-type scale ranging from 0 (not at all) to 5 (a great deal). Since debriefing was not required by the IRB or Pollfish for this study, the participants were simply reminded that they are to contact the first author with any questions or concerns about the survey.

## 5. Study 2: Results and Discussion

An analysis of covariance (ANCOVA) was conducted to compare the scores between the two experimental conditions on Coronavirus Asiaphobia, controlling for the degree to which: (a) participants’ or their loved ones’ health has been impacted by Coronavirus; (b) the financial well-being of the participants or their loved ones has been adversely affected by the pandemic; and (c) the participants regularly interact with their AAPI friends, neighbors, or colleagues.

Participants in the condition in which envy toward AAPIs was induced (Condition 1) scored significantly higher on the Coronavirus Asiaphobia scale than their counterparts in the condition in which generalized envy was induced (Condition 2), Ms (SDs) = 16.76 (5.91) versus 14.20 (6.76), respectively, with higher scores indicating more Asiaphobic responses (F(4) = 7.98, *p* < 0.0001). As hypothesized, therefore, participants in the condition in which envy toward AAPIs was induced through biographical recall tended to score higher on the Asiaphobia scale compared to their counterparts who were in the condition in which general envy was induced without targeting any social groups. It can therefore be concluded that envious prejudice toward AAPIs fueled Asiaphobia in the context of Coronavirus. As was the case with Study 1, analyses comparing the demographic groups were not performed for the reasons previously discussed, involving the skewed distribution across the groups and, more importantly, the lack of a theoretical basis to suspect such differences according to the stereotype content model.

**Figure 1:**
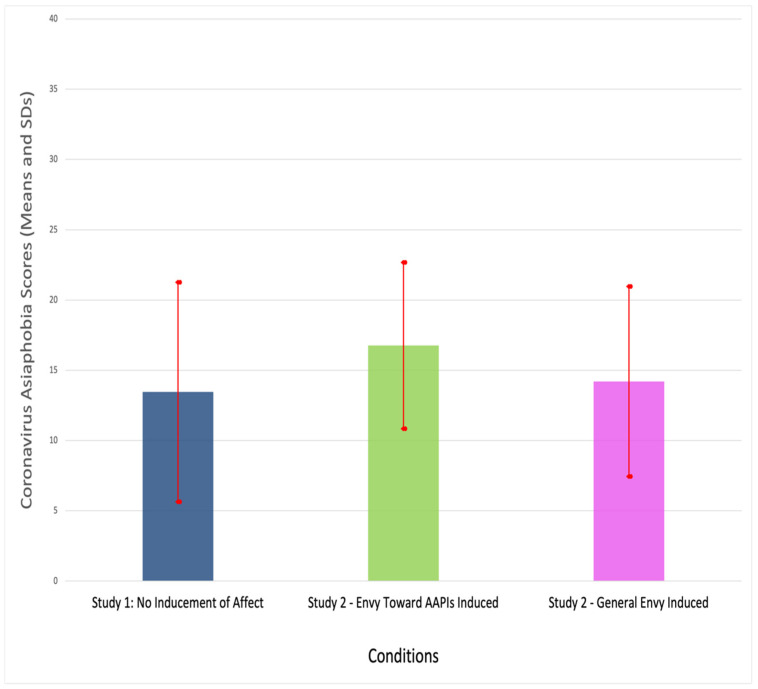
Mean Coronavirus Asiaphobia scores across three conditions and corresponding standard deviations.

It is interesting to note here that the Coronavirus-related Asiaphobia scores obtained in Study 2 seem generally higher than what was obtained in Study 1 (i.e., M = 13.46; SD = 7.81). A one-way ANOVA comparing the three means—and three separate groups of participants—across two studies with a post hoc test (Tukey’s HSD, *p* < 0.005) reveals a curious overall outcome as summarized in Table 5 (F(2, 304) = 5.685, *p* < 0.004) and illustrated in Figure 1 (a one-way ANOVA was performed here without controlling for the magnitudes of health and financial impact the pandemic has had or the extent of contact participants have with AAPIs, as these variables were not included in Study 1).

Namely, the condition in which envy toward AAPIs was induced (Study 2, Condition 1) yielded significantly higher Coronavirus Asiaphobia scores than those in the “general envy” condition (Study 2, Condition 2) or participants who did not receive any experimental inducement of affect (Study 1). This further strengthens the hypothesized notion that envious attitude toward AAPIs serves to trigger the pandemic-related anti-AAPI sentiments.

## 6. Overall Discussion

This research has demonstrated that, consistent with what the stereotype content model [26] suggests, envy plays an important role in understanding Coronavirus-related Asiaphobia. Study 1 demonstrated that scores on the short version of Lin and her colleagues’ [27] SAAAS, which measures envious stereotyping toward AAPIs, were predictive of Coronavirus Asiaphobia. More compellingly, Study 2 demonstrated that, when envy toward AAPIs was experimentally induced, participants tended to score higher on the Coronavirus Asiaphobia measure, thereby causally establishing the relationship between envy toward AAPIs and Coronavirus Asiaphobia.

Additionally, the results across the two studies reveal that the Coronavirus Asiaphobia scores were statistically higher among the participants among whom envy toward AAPIs was induced compared to their counterparts who either experienced inducement of envy toward unspecified ethnic groups or did not experience any experimental inducement of emotions. It can hence be concluded that envy toward AAPIs seemingly prompts individuals to express Asiaphobia related to the pandemic. This assertion is consistent with the hypothesis based on the stereotype content model, whereby envious prejudice against an outgroup has been shown to lead to xenophobia, marked by dehumanization, scapegoating, or even schadenfreude [26,27,28,29,30].

Given these results, what can we do to mitigate the reported surge in Asiaphobia and hate incidents during the pandemic? First, since the current results signal that the affect of envy serves as the catalyst for Coronavirus-related Asiaphobia, it is reasonable to characterize Asiaphobia in the current context to be primarily affect based. As such, affectively based messages would be more effective in alleviating Asiaphobia than would cognitively based messages [38]. Keeping in mind that envious Asiaphobia, according to the stereotype content model, primarily consists of dehumanized perceptions of AAPIs being competent—as signaled by such ostensibly desirable dimensions as academic, professional, and financial success—and of a lack of human warmth. Affectively laden messages that showcase the sides of AAPIs that would counter these construals would therefore be good candidates to fight Asiaphobia. In that sense, it may be important for AAPIs to communicate openly and widely about the fears, anxieties, stresses, and other difficulties that they have been experiencing as AAPIs during the current pandemic.

While the current research has established a causal relationship between envious prejudice toward AAPIs and Coronavirus-related Asiaphobia based on the stereotype content model [26,27,28,29,30], additional research is needed to rule out the possibility that the observed effect here may not be unique to envy and, instead, other affects may produce similar results. This concern may be particularly relevant in demystifying Asiaphobia during the current pandemic, during which many Americans grapple with a wide range of restrictions, hardships, and other challenges along various domains, ranging from health, finances, education, to family. During such turbulent times, a closer examination of affective dimensions for understanding Asiaphobia may be useful, although such a prediction may not necessarily be warranted based on the stereotype content model.

Secondly, in Study 2, in which envy was induced, it should be noted that the experimental manipulation was temporary at best, and it could be argued that the inducement of envy, in and of itself, does not closely emulate the envious prejudice that some individuals hold on a more chronic basis, as defined in the stereotype content model (see [30]). Thus, further research would be needed to clarify some of the potential conflation in order to further expand our understanding of Coronavirus-related Asiaphobia. Such nuanced understanding can be achieved through additional research, delineating various affective dimensions pertaining to envy toward an outgroup.

Third, on a more technical note, in this exploratory research, we have not invested very much effort into detailing the development and validation of the scales used to assess envious prejudice or Coronavirus-related Asiaphobia. Instead, we rely primarily on McDonald’s omega as an indicator of reliability for the scales that we have utilized. Additional efforts that probe more rigorously into the scale development and validation processes may thus be in order if we are to further investigate the relationship between envy and Asiaphobia more comprehensively.

Finally, given the notoriously modest relationship between attitudes and behavior [39], it would be useful for future research to examine the extent to which Coronavirus-related Asiaphobia may manifest itself in anti-AAPI hate incidents during the current pandemic. With the advances in the experimental methods that would permit researchers to simulate interpersonal and intergroup behavior virtually, for instance, it may be feasible to design an experiment in which envy toward AAPIs is induced and participants, subsequently, are given potential opportunities to act in an anti-AAPI manner. Such a study would provide a more direct insight into the recent spike in anti-AAPI hate incidents.

As we enter the third year of the current global pandemic, in the U.S., AAPIs continue to face Coronavirus-related Asiaphobia, and concerns regarding its implications for the psychological health of AAPI children and adults have been widely raised [2,7,8]. In fact, empirical evidence has begun surfacing in support of such apprehensions, urging us to take Asiaphobia as an area of urgent priority [6]. The studies reported here represent preliminary results that provide clues as to how our society may be able to mitigate Coronavirus-related Asiaphobia, focusing primarily on the envious ambivalence that some individuals hold toward AAPIs. Taking the current global crisis, which has claimed millions of lives, as an opportunity for facilitating harmonious intergroup relationships, further research should be conducted to better understand the nature of prejudice and xenophobia, as they manifest themselves in a unique set of circumstances as those we currently face, and beyond.

## Figures and Tables

**Table 1:** Demographic characteristics of the participants included in Study 1 (N = 140).

Categories	Ns
**Age**	
18–24	9
25–34	37
35–44	54
45–54	17
55–64	23
**Sex**	
Females	79
Males	61
**Race/Ethnicity**	
White	107
Black	21
Latin	12

**Table 2:** The Scale of Anti-Asian American Stereotypes—15-Item Short Version, adopted from Lin, Kwan, Cheung, and Fiske (**ω** = 0.86).

On a Likert-Type Scale from 0 (Strongly Disagree) to 5 (Strongly Agree):
1 (R) As a group, Asian Americans are not constantly in pursuit of more power.
2 (R) Asian Americans are a group not obsessed with competition.
3 Oftentimes, Asian Americans think they are smarter than everyone else is.
4 Asian Americans seem to be striving to become number one.
5 Asian Americans are motivated to obtain too much power in our society.
6 Many Asian Americans always seem to compare their own achievements to other people’s.
7 When it comes to education, Asian Americans aim to achieve too much.
8 In order to get ahead of others, Asian Americans can be overly competitive.
9 Asian Americans commit less time to socializing than others do.
10 Asian Americans do not usually like to be the center of attention at social gatherings.
11 (R) Asian Americans put high priority on their social lives.
12 Most Asian Americans are not very vocal.
13 Asian Americans do not interact with others smoothly in social situations.
14 Asian Americans are not as social as other groups of people.
15 (R) Asian Americans spend a lot of time at social gatherings.

(R): Reverse scored.

**Table 3:** The Coronavirus Asiaphobia Scale (**ω** = 0.76).

On a Likert-Type Scale from 0 (Strongly Disagree) to 5 (Strongly Agree):
1 (R) Anti-Asian discrimination during the Coronavirus pandemic is a major social problem.
2 Asians in the U.S. pose Coronavirus risks.
3 (R) I find it offensive to refer to Coronavirus as the “China virus” or “Chinese virus.”
4 Asian people are probably responsible for the current pandemic.
5 (R) I’ve heard quite a bit about some people physically assaulting or verbally attacking Asian Americans during the pandemic.
6 I would worry about having physical contacts with Asians (more so than with other groups) because of the potential risk of Coronavirus infection.
7 (R) Asian Americans seem to get blamed for Coronavirus.
8 I can understand why some people would be upset with Asian people for the Coronavirus pan-demic.

(R): Reverse scored.

**Table 4:** Demographic characteristics of the participants included in Study 2 (N = 167).

Categories	Ns
**Age**	
18–24	25
25–34	34
35–44	65
45–54	21
55–64	22
**Sex**	
Females	
Males	81
**Race/Ethnicity**	
White	140
Black	13
Latinx	14

**Table 5:** Comparison of the mean Coronavirus Asiaphobia scores across two studies.

Conditions	Means (SDs)
Study 1: No inducement of affect (N = 140)	13.46 (7.81) ^a^
Study 2-Condition 1: Envy toward AAPIs induced (N = 80)	16.76 (5.91) ^b^
Study 2-Condition 2: Target-unspecific envy induced (N = 87)	14.20 (6.76) ^a^
F(2, 304) = 5.685, *p* < 0.004; ^b^ > ^a^	

Differences in letters represent statistically significant differences at *p* < 0.005, Tukey’s HSD.

## Data Availability

The data presented in this study are available on request from the corresponding author. The data are not publicly available due to an IRB clause limiting open dissemination of unaggregated data.
